# Phytochemical profiling and bioactive potential of bael gum using UHPLC-Q-TOF-MS: a novel nutraceutical source

**DOI:** 10.3389/fnut.2025.1720060

**Published:** 2025-12-17

**Authors:** Suchisree Jha, Ashok Yadav, Saran Kumar Gupta, Asha Ram, Girija Choudhary, Sandeep Garg, Hirdayesh Anuragi, Ronak Yadav, Guntukogula Pattabhi Sandeep, Prasad Manikrao Sonwalkar, Naresh Kumar, Inder Dev

**Affiliations:** 1 Indofil Industry Ltd., Thane, Maharashtra, India; 2 ICAR-Central Agroforestry Research Institute, Jhansi, Uttar Pradesh, India; 3 Department of Botany, Kalimpong College, Kalimpong, West Bengal, India; 4 Department of Biotechnology, Bundelkhand University, Jhansi, Uttar Pradesh, India; 5 Department of Horticulture, Bundelkhand University, Jhansi, Uttar Pradesh, India; 6 Department of Fruit Science, Rani Lakshmi Bai Central Agricultural University, Jhansi, Uttar Pradesh, India; 7 Dr Yashwant Singh Parmar University of Horticulture and Forestry, Nauni, Himachal Pradesh, India

**Keywords:** antidiabetic, antioxidant, bael, ethnomedical, gum, nutraceutical

## Abstract

**Introduction:**

Plant gums are recognized for their notable nutraceutical and medicinal properties. Numerous studies have explored the medicinal potential of *Aegle marmelos*, particularly leaves, fruits, and seeds. Its hardy nature, drought tolerance, and diverse applications make it a promising climate-smart crop for agroforestry systems.

**Methods:**

The present study was therefore designed to address this limitation by conducting an in-depth phytochemical and nutraceutical profiling of bael gum and evaluating different parameters, i.e., macroscopic parameters, physical parameters, antioxidant enzyme activities, total phenol content, antioxidant analysis (DPPH assay, metal chelating activity, and ABTS assay). Ultra-high Performance Liquid Chromatography coupled with Quadrupole Time-of-Flight Mass Spectrometry (UHPLC-QTOF-MS) was used to identify a diverse range of bioactive compounds with potential health benefits.

**Results:**

This study presents a detailed nutritional and phytochemical characterization of bael gum, revealing high levels of total polysaccharides (40.94%), total protein (16.2%), total nitrogen (2.59%), and total amino acids (16.14%). The antioxidant assays demonstrated significant activity with IC₅₀ values of 765.7 ± 1.5 mg/g for metal chelation, 488 ± 1 mg/g for DPPH, and 368.7 ± 0.9 mg/g for ABTS, alongside of total phenol content of 90 ± 1 μg/g FWT.

**Conclusion:**

These findings revealed that bael gum is a valuable source of bioactive compounds with promising therapeutic potential and its applicability across multiple industries, including food and beverage, pharmaceutical, cosmetic, and personal care, as well as biodegradable films and edible packaging materials. Furthermore, investigating the mechanisms of action of individual bioactive constituents and evaluating their effectiveness in functional food and therapeutic formulations will be essential to enhance the practical utilization of bael gum.

## 1 Introduction

Bael (*Aegle marmelos* L.) is a highly valued tree species among the 0.25 million plant species available on Earth. In different languages, the bael tree has various names, i.e., Bel (Assamese, Bengali & Urdu), Bel, Maredu (Marathi), Bel (Hindi), Heirikhagok (Manipuri), Vilvam (Malayalam), Sandiliyamu (Telugu), Bilvapatre (Kannada), Bello (Konkani), Bili, Bilipatr (Gujarati), Bel-thei (Mizo), Bengal quince, Beli fruit, golden apple, and stone apple (English), Adhararuha, Tripatra, Bilva/Shivaphala, Sivadrumah (Sanskrit), Belo (Oriya), and Vilva Marum (Tamil).^1^ The diversity of names reflects its wide distribution and cultural significance across different parts of India (1, 2). Additionally, in areas where Hindus reside, it is revered by worshippers and frequently planted next to temples devoted to Lord Shiva (3). Since ancient times, bael has been highly valued and widely used in Ayurvedic medicine, both in India and across the South Asian region (4).

The bael tree is believed to have originated in Central India and the Eastern Ghats and is considered native to Southeast Asia. It thrives in tropical to subtropical climates and is commonly found growing wild up to 500 meters above mean sea level (m asl) in the lower Himalayan hills. Naturally occurring populations of bael are found in the Himalayan foothills, particularly in Uttarakhand and the lower districts of Himachal Pradesh, as well as in Central India (Chhattisgarh, Madhya Pradesh, and Uttar Pradesh), the Deccan Plateau, and along the eastern coastal regions of India. The bael tree is deciduous in nature. Its fruits are spherical with a hard, woody shell (grey or yellowish) and contain soft, yellow to orange, mucilaginous pulp (5).

Bael fruit offers a wide range of health benefits due to its rich content of minerals, fatty acids, amino acids, carbohydrates, vitamins, dietary fibers, and phytochemicals (6). Moreover, it is traditionally used in diabetes, cardiovascular disorders, inflammatory conditions, and digestive ailments. Scientific studies have demonstrated the fruit’s protective properties against radiation, microbial infections, oxidative stress caused by free radicals, wounds, and even symptoms of depression. These findings highlight the significant natural healing potential of bael. In Ayurveda and other traditional systems of medicine, every part of the bael tree —root, bark, leaves, flowers, and fruit —is valued for its therapeutic properties in treating various diseases. Numerous studies have been conducted on various parts of the bael plant, revealing a wide spectrum of ethnopharmacological activities. These include antimicrobial, anti-diarrheal, anti-diabetic, anti-hyperlipidemic, antifungal, antibacterial, anti-ulcer, anticancer, cardioprotective, antipyretic, anti-inflammatory, radioprotective, hepatoprotective, anti-spermatogenic, wound healing, anticonvulsant, immunomodulatory, antidepressant, anti-genotoxic, anti-proliferative, antimalarial, anti-microfilarial, anti-arthritic, nephroprotective, anti-thyroid, insecticidal, and anti-ocular hypertensive effects (2, 7, 8).

Plant gums are secreted from various parts of the plant, including stems, branches, twigs, leaves, and bark, and can also originate from the seed epidermis (9). Gums produced from plants are extremely valuable items that are valued for their medical applications, bioavailability, and nutritional qualities. Gums derived from plants have been utilized for a wide range of purposes throughout history. In modern times, they are extensively applied in the food industry for diverse functions, including as microencapsulating agents for flavor and color, prebiotics, thickeners, beverage stabilizers, coatings, emulsifiers, fat replacers, clarifying agents, edible packaging materials, and in the formulation of emulsions (10). Plant-derived gums have long served a variety of purposes and are increasingly recognized as valuable resources in pharmaceutical applications. Their biocompatibility, nutritional richness, edibility, cost-effectiveness, and therapeutic potential make them highly suitable for modern drug formulations. These natural polymers improve patient compliance, reduce adverse effects, and are well-tolerated by the skin and eyes without triggering allergic reactions. Their low production cost further contributes to their growing utility in the pharmaceutical sector (11). In traditional systems of medicine, bael gum has been employed for a range of therapeutic purposes (12). It is primarily utilized as a gelling agent, adhesive, waterproofing compound, and a controlled drug release carrier (13) and has antimicrobial and anticoagulant properties (14).

The ethnopharmacological significance of plant gums has long been recognized, yet their comprehensive investigation remains limited. Although numerous studies have explored the medicinal potential of *Aegle marmelos*, particularly leaves, fruits, and seeds, there is a notable lack of detailed information regarding the nutraceutical and phytochemical attributes of bael gum. These gaps in knowledge restrict the understanding of the full therapeutic potential of the plant. The present study was therefore designed to address this limitation by conducting an in-depth phytochemical and nutraceutical profiling of bael gum. A key objective was to identify and characterize its bioactive constituents and to explore potential correlation with bioactive constituents previously reported in other plant parts, thereby contributing to a more holistic understanding of its pharmacological value.

## 2 Materials and methods

### 2.1 Sampling of bael gum

Bael gum was harvested from the bael plants in Babina block, Jhansi district, Uttar Pradesh. The naturally exuded gum was manually harvested from twigs, branches, and stems. It was initially subjected to physical cleaning to remove adhering bark and extraneous matter. To attain the required brittleness, the collected gum was first air-dried for 120 h and then dried again in a hot air oven set at 50 °C. Once dried, the gum was ground using a high-speed electric blender (Bajaj, India) and sieved through a 100-mesh screen to obtain a uniform powder. To prepare the gum extract, the powdered gum was mixed with distilled water in a 1:10 (w/v) ratio. The mixture was stirred continuously at room temperature for 24 h using a rotary shaker to facilitate gum solubilization. The resulting solution was filtered through muslin cloth to separate the supernatant. The residual material retained on the cloth was thoroughly rinsed with distilled water, and the washings were combined with the original filtrate to ensure complete recovery. This extraction procedure was repeated three times to maximize yield (15).

### 2.2 Separation and purification process

**Figure 1:**
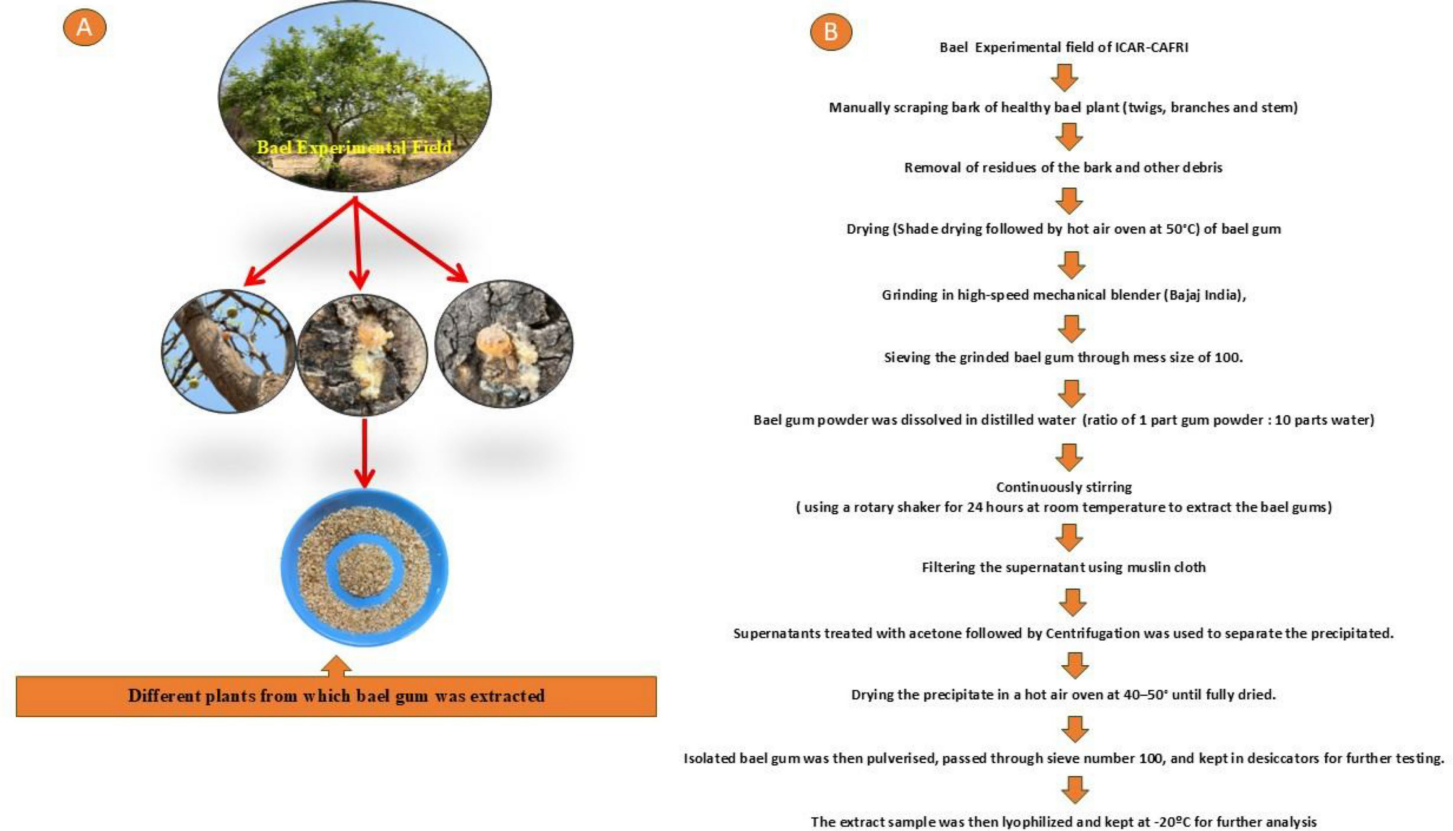
**(A)** Collection of bael gum from the experimental field of bael orchard. **(B)** Flow chart for sample preparation of bael gum.

Bael gum was isolated from the previously obtained supernatant by precipitation using acetone at a 2:1 (v/v) ratio. The precipitated gum was separated via centrifugation and subsequently dried in a hot air oven at 50 °C. The dried material was then finely ground and passed through a 100-mesh sieve to ensure uniform particle size. The purified gum was stored in desiccators before further analysis. The isolated sample was then lyophilized and stored for further examination at −20 °C (Figure 1).

### 2.3 Macroscopic parameters

Various macroscopic features like color, flavor, shape, surface appearance, texture, and fractures were systematically examined. These attributes were categorized using predefined classification schemes based on odour, taste, and texture. The mechanical properties of the gum, including viscosity, adhesiveness, elasticity, hardness, and geometric attributes, were evaluated through various analytical techniques. Taste was assessed using a five-point hedonic scale (1: sweet, 2: sour, 3: salty, 4: bitter, and 5: umami), while odor intensity was measured on a seven-point scale: no odor, very weak, weak, distinct, strong, very strong, and intolerable (16–18).

### 2.4 Physical parameters

The physical properties of bael gum analyzed in this study included nitrogen content, amino acid composition, polysaccharide concentration, protein levels, electrical conductivity, pH, and viscosity. Viscosity measurements were performed at 30 °C using rotational speeds of 50 rpm and 100 rpm, expressed in mPa·s. Moisture content was determined using a Contech moisture meter, while pH was recorded with a calibrated pH meter. Electrical conductivity was measured with an EC meter and expressed in Siemens per meter (S/m). Viscosity was further assessed using an Ostwald viscometer. Total polysaccharide (phenol sulfuric acid method) (19, 20), total protein (21) total N (22), and the amino acid % (23) were estimated by standard protocols.

### 2.5 Antioxidant enzyme activities

Using modified protocols, different antioxidant enzymes in bael gum were quantified, including polyphenol oxidase (PPO) (24), catalase (25), ascorbate peroxidase (26), NADPH oxidase (27) superoxide dismutase (28), GST (29) and glutathione reductase (30). The extinction values (*ε*) utilized for enzymes such as catalase, guaiacol peroxidase, glutathione reductase, and ascorbate peroxidase were 2.8, 26.6, 6.22, and 2.8 mM^−1^ cm^−1^, respectively. The UV–VIS spectrophotometer absorbance readings were 240, 470, 560, 340, and 290 nm for catalase, guaiacol peroxidase, superoxide dismutase, glutathione reductase, and ascorbate peroxidase, respectively.

### 2.6 Antioxidant analysis

The antioxidant analysis, such as total phenol content (TPC) (31, 32), DPPH assay, metal chelating activity (31, 33), and ABTS assay (31, 32) were performed as per the standard protocols. Absorbance readings were measured at 517, 734, 562, and 290 nm for DPPH, ABTS^+^, Metal chelating, and TPC, respectively, using a UV–VIS spectrophotometer. Detailed methodologies for each antioxidant assay are provided below.

The details of different antioxidant analyses is mentioned below.

#### 2.6.1 Total phenolic content (TPC)

The total phenolic content of bael extract was measured according to the standard protocol (31, 32). 1 mL of the extract was reacted with a mixture containing 1 mL of 95% ethanol solution, 5 mL of distilled water, and 500 μL of Folin-Ciocalteau reagent (50%). After an incubation period of 5 min, 1 mL of 5% Na_2_ CO_3_ was added. It was mixed thoroughly on a vortex shaker and further kept for 1 h of incubation at room temperature. Finally, the absorbance of the coloured reaction mixture was recorded at 765 nm against the reagent blank.

#### 2.6.2 1,1-diphenyl-2 picrylhydrazyl (DPPH) based free radical scavenging activity

The radical scavenging activity of the bael extract was measured by DPPH method (31, 33). The reaction mixture contained 1.8 mL of 0.1 mM DPPH and 0.2 mL of bael extract. The reaction mixture was vortexed and left in the dark at room temperature for 30 min. The absorbance was measured at 517 nm. A reaction mixture without test sample was considered as control. Radical scavenging activity was expressed as percent inhibition from the given formula:

Percentage inhibition of DPPH radical = [(A_0_ – A_1_)/A_0_] x 100.

Where, A_0_: absorbance of the control and A_1_: absorbance of the extract.

#### 2.6.3 Metal chelating activity

The chelating activity of the extracts for ferrous ions Fe^2+^ was measured according to the method (31, 33). 0.4 mL of extract was mixed with 0.04 mL of FeCl_2_ (2 mM) solution. After 30s, 0.8 mL ferrozine (5 mM) solution was added. After 10 min at room temperature, the absorbance of the Fe^2+^–Ferrozine complex was measured at 562 nm. The chelating activity of the extract for Fe^2+^ was calculated as

Chelating rate (%) = (A_0_ - A_1_)/A_0_ × 100

Where A_0_ was the absorbance of the control (blank, without extract) and A_1_ was the absorbance in the presence of the extract.

#### 2.6.4 2,2-azinobis-(3-ethylbenzothiazoline-6-sulfonic acid) diammonium salt (ABTS^+^) radical cation(s) decolorization assay

The spectrophotometric analysis of ABTS^+^ radical cation(s) scavenging activity was measured according to Jha et al. (31) and Re et al. (32). This method is based on the ability of antioxidants to quench the ABTS^+^ radical cation, a blue/green chromophore with characteristic absorption at 734 nm. The ABTS^+^ was obtained by reacting 7 mM ABTS^+^ radical cation(s) in H_2_O with 2.45 mM potassium persulfate (K_2_S_2_O_8_), stored in the dark at room temperature for 6 h. Before usage, the ABTS^+^ solution was diluted to get an absorbance of 0.750 ± 0.025 at 734 nm with sodium phosphate buffer (0.1 M, pH 7.4). Then, 2 mL of ABTS^+^ solution was added to 1 mL of the bael extract. After 30 min, the percentage inhibition at 734 nm was calculated for each concentration, relative to a blank absorbance. Solvent blanks were run in each assay. The inhibition of the ABTS radical (%) was calculated using a similar equation to that for the DPPH method.

### 2.7 Antidiabetic assay

The *α*-glucosidase and *α*-amylase inhibitory properties of bael gum were assessed to evaluate its antidiabetic properties, as per standard procedures (34, 35).

#### 2.7.1 *In vitro* α-glucosidase inhibitory activity

The *α*-glucosidase inhibitory property of the sample extract was assayed as described by Sudha et al. (36). The different concentrations of extract were prepared by adding 0.2 mM phosphate buffer (pH 6.8). After that, 0.1 mL of enzyme solution was added and kept for incubation at 37 °C. Then, 0.25 mL pNPG (p-Nitrophenyl-glucopyranoside) (3 mM) was added, and the reaction was terminated by adding 4 mL of Na_2_ CO_3_ (0.1 M). The *α*-Glucosidase inhibition activity was estimated by determining the kinetics of release of pNPG (p-Nitrophenyl-glucopyranoside) at 405 nm. The control contained all the reagents without the sample extract. The *α*-glucosidase inhibitory activity was estimated by following equation:

Inhibitory ratio % = [1- (As- Ab)/ Ac] × 10where As, Ab and Ac represent the OD value of the sample, blank, and control reaction mixture, respectively.

#### 2.7.2 *In vitro* α-amylase inhibitory activity

The α-amylase inhibition potential of the extract was estimated by standard spectrophotometric method (37). Briefly, 0.5 mL of bael extract was mixed with 0.5 mL of α-amylase solution and incubated at 37 °C for 5 min. After the initial incubation, 0.5 mL of 1% starch solution was added to the reaction mixture and incubated further for 10 min. To terminate the enzymatic reaction, 1 mL of dinitrosalicylic acid (DNSA) reagent was added, followed by heating the mixture in a boiling water bath for 10 min until the solution developed an orange-red color, indicating the presence of reducing sugars. The reaction mixture was then cooled to room temperature and diluted to a final volume of 5 mL with distilled water. The optical density (OD) was recorded at 540 nm using a spectrophotometer. The *α*-amylase inhibitory activity was quantified by calculating the concentration of the sample extract required to inhibit 50% of the enzyme activity (IC₅₀).

### 2.8 UHPLC-QTOF-MS analysis of bael gum

Bael gum was further analyzed using the UHPLC-QTOF-MS technique as per the procedure described by Haron et al. (38). Metabolite profiling was carried out using G6550A Ion Mobility Q-TOF LC/MS (Agilent, Santa Clara, USA) on the Agilent 6,500 Series Q-TOF LC/MS System (version B.05.01, B5125.3), which combines quadrupole and TOF analyzers for high-resolution and accurate mass detection. Analyte separation was achieved using a Hypersil Gold column (100 × 2.1 mm, 3 μm, Thermo Fisher Scientific, Mumbai, India). The gradient elution method consisted of mobile phases water (0.1% formic acid) as phase A and acetonitrile (0.1% formic acid) as phase B. The steep gradient elution for the analysis of bael gum was carried out with the following procedure: 0–1.0 min at 5 percent of B, 1.1–20.0 min at 100 percent of B, 20.1–25.0 min at 100 percent of B, 25.1–26.0 min at 5 percent of B, and 26.1–30.0 min at 5 percent of B. The column was kept at 40 °C, the injected sample volume was set at 5.0 μL, and the elution flow rate was set at 0.3 mL/min (17). Thermo Scientific’s Xcalibur (version 4.2.28.14) and Agilent MassHunter Workstation Software (version B.05.01) were used in conjunction with Compound Discoverer 3.2 SPI software to acquire and process the data. Minor modifications were applied to optimize the method for natural product analysis. After each cycle, the column was flushed for 2 min to prepare for the subsequent injection. The highest flow rate for ramp-up and ramp-down was set at 100.0 mL/min^2^, with pressure ranging from 0 to 1,200 bar. Agilent MassHunter Workstation Software (version B.05.01, B5125.3) was used to process and analyze the collected data on some bael gum parameters. Agilent Mass Hunter Workstation Software-Data Acquisition for 6550A Series Q-TOF, version B.05.01 (B5125.3), was used for data processing and analysis. The default parameters were slightly modified to better support the analysis of natural products.

Library matching, elemental composition determination, feature recognition, background subtraction utilizing blank data, retention time alignment, and fragmentation search (FISh) score were all part of the procedure. Chemical constituents in bael gum were identified primarily through comparison of MS/MS data with the mzCloud database. For unmatched signals with a FISh score above 40, identification was reattempted using the ChemSpider database based on MS data.

### 2.9 Statistical analysis

The present study profiled bael gum using samples collected through random sampling, which provided an overall understanding of its biochemical and nutraceutical attributes. XLSTAT software and Microsoft Excel were used to analyze all of the data using ANOVA analysis. The mean value of three replications is used to characterize the results.

## 3 Results and discussion

### 3.1 Macroscopic parameters

Table 1 presents an analysis of the morphological traits of bael gum. It exhibits a pale golden to yellowish hue, which is visually appealing. The gum has a firm texture, a salty taste, and is odorless. It also possesses a viscous and thick consistency. Gum particles are crystalline in form and structure, and they are categorized as gritty, grainy, and coarse. These morphological traits are a strong indicator of the gum’s quality and contribute to its potential marketability and versatility in various industries, particularly in the pharmaceutical sector. Similar high-quality appearances have also been reported in other gums, such as gum Arabic (39), Chironji gum (17), almond gum (40) and olibanum gum (34).

**Table 1:** Morphological parameters analysis of bael gum.

S. No.	Morphological parameters	Properties
1	Color	Light transparent white
2	Odour	No odour
3	Taste	Salty
4	Texture hardness	Hard
5	Viscosity	Thick and viscous
6	Elasticity	Plastic
7	Adhesiveness	Sticky
8	Particle size	Gritty, grainy, and coarse
9	Particle shape and orientation	Crystalline
10	Surface appearance	Smooth

### 3.2 Physical attributes

Table 2 summarizes the physical characteristics of bael gum. The gum exhibits a moisture content of 8.9% and an average pH of 6.55 in a 1% aqueous solution. It is rich in total polysaccharide (40.94%), total protein (16.2%), total nitrogen (2.59%), and total amino acid (16.1%). Bael gum also demonstrates a conductivity of 860 S/m and shows good solubility (89.0%) in warm water. Viscosity measurements indicate that a 1% solution of bael gum has a viscosity of 605 mPa·s at 30 °C and 50 rpm, which decreases to 345 mPa·s at 100 rpm, suggesting shear-thinning behavior.

**Table 2:** Physical attributes and antioxidant enzyme analysis of bael gum through different methods.

Attributes		Bael gum
Physical attributes	Moisture %	8.90 ± 0.80
	pH (1% solution)	6.55 ± 0.52
	Total Polysaccharide (%)	40.94 ± 1.06
	Total Protein (%)	16.20 ± 1.03
	Total Nitrogen (%)	2.59 ± 0.18
	Total Amino acid (%)	16.10 ± 1.16
	Conductivity (S/m)	860 ± 6.50
	Viscosity of 1% solution (mPa.s) a 30 °C at 50 rpm	605 ± 2.00
	Viscosity of 1% solution (mPa.s) a 30 °C at 100 rpm	345 ± 4.50
Antioxidant enzymes	POD: Peroxidase (U/min/g)	1.91 ± 0.16
	PPO: Polyphenol Oxidase (U/min/g)	1.93 ± 0.21
	CAT: Catalase (U/min/g)	6.85 ± 0.48
	NOX: NADPH oxidase (U/min/g)	0.42 ± 0.037
	SOD: Superoxide dismutase (U/min/g)	1.56 ± 0.11
	APX: Ascorbate peroxidase (U/min/g)	2.32 ± 0.18
	GST: Glutathione S-transferases (U/min/g)	2.08 ± 0.16
	GR: Glutathione reductase (U/min/g)	0.24 ± 0.006

These properties are comparable to those observed in gums derived from other plant sources such as gum acacia (*Acacia senegal*), axlewood (*Anogeissus latifolia*), thorny acacia/babul (*Acacia nilotica*), and Indian tragacanth (*Sterculia urens* Roxb) (35). According to Zhou et al. (41)protein plays a vital role in maintaining human health, and the protein content of the bael gum was 16.2%. Other plant gums, such as khaya gum, chironji gum, and honey locust gum, have also been reported to contain appreciable amounts of protein (10, 17). These findings highlight the potential of bael gum as a valuable dietary component.

### 3.3 Antioxidant enzyme activities

**Figure 2:**
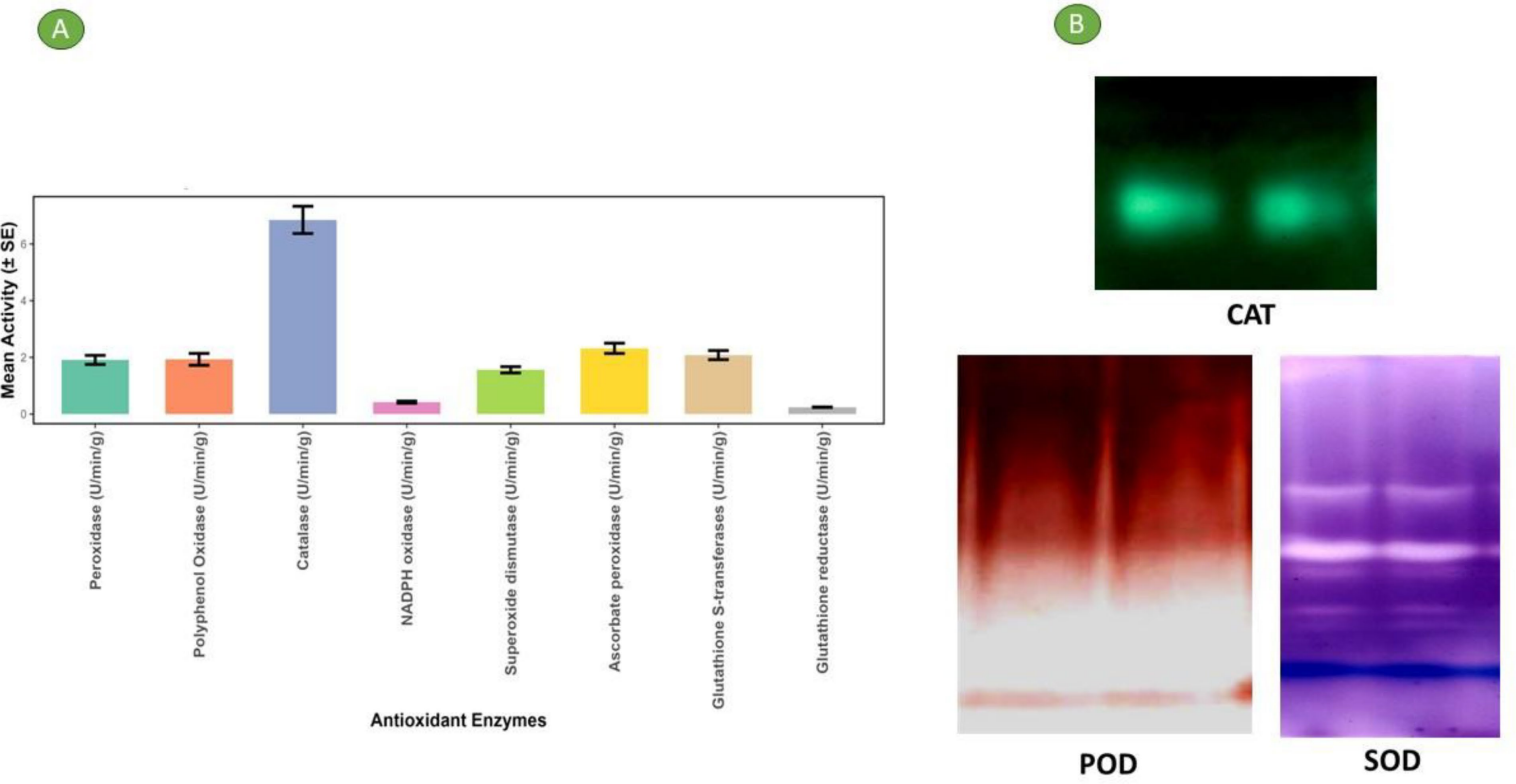
**(A)** Antioxidant enzyme activity through different methods in the bael gum. **(B)** Gel picture of different antioxidant enzyme content in bael gum.

Antioxidant enzymes in plants are crucial for defending plant cells from oxidative injury caused by exposure to environmental stressors like UV radiation, pollution, pathogens, and adverse weather conditions. In addition to their protective functions, these enzymes are also involved in regulating key physiological processes, including plant growth and development, responses to both biotic and abiotic stress. Maintaining a balanced antioxidant enzyme system is essential for plant health and resilience (42). Table 2 and Figure 2 present the various antioxidant enzyme activities of bael gum. Superoxide dismutase (SOD), which mitigates the harmful effects of superoxide radicals by catalyzing their conversion into molecular oxygen and hydrogen peroxide, was found to have an activity of 0.76 U/min/g (43). Catalase decomposes H_2_O_2_ (hydrogen peroxide) into water and oxygen, preventing oxidative injury to cellular structures (44), and its activity was recorded as 6.85 U/min/g. Peroxidases, such as glutathione peroxidase (GPX) and ascorbate peroxidase (APX), utilize reducing agents like ascorbate and glutathione to detoxify hydrogen peroxide and organic hydroperoxides (45). The peroxidase (POD) activity in bael gum was recorded at 1.91 U/min/g. Glutathione reductase (GR), which regenerates reduced glutathione—a key antioxidant—by converting its oxidized form back to its active state, showed an activity of 0.24 U/min/g (46). Similarly, Chironji gum has been reported to contain significant levels of antioxidant enzymes, including CAT, GPX, SOD, GR, and APX, further supporting the biochemical relevance of plant-derived gums in oxidative stress management (17).

### 3.4 Antioxidant analysis

The antioxidant activity of plant extracts is being assessed to estimate their medicinal value, because antioxidants play a vital role in scavenging free radicals and preventing diseases associated with oxidative stress (47). Therefore, DPPH, ABTS, and metal chelation assays were used in the current investigation to assess the bael gum’s antioxidant activity. The DPPH assay is a widely used, reliable, and sensitive method for evaluating the antioxidant activity of plant extracts. It is valued for its simplicity and rapid execution, although its effectiveness may vary depending on the plant source and extraction procedure (48). The assay measures the ability of antioxidants to scavenge DPPH free radicals, which is observed spectrophotometrically at 517 nm. The antioxidant strength was commonly expressed as IC_50_. In the present study, bael gum extract exhibited notable antioxidant activity, with IC₅₀ values of 488.3 ± 1 mg/g for the DPPH assay and 368.7 ± 1 mg/g for the ABTS assay, indicating that the extract was more effective in scavenging ABTS free radicals compared to DPPH (Table 3).

**Table 3:** Antioxidant analysis of bael gum through different methods.

S. No.	Therapeutic potential	Methods	Quantity
1	Antioxidant assay	DPPH Assay (IC50 mg/g)	488.25 ± 0.97
		Metal Chelating (IC50 mg/g)	765.70 ± 1.53
		ABTS (IC50 mg/g)	368.65 ± 0.92
		Phenol (TPC ug/g FWT)	90.30 ± 1.30
2	Antidiabetic activity	*α-Amylase* (μg/mL)	1126.45 ± 18.22
		*α-Glucosidase* (μg/mL)	852.97 ± 14.86

Another crucial antioxidant mechanism is the ability of certain compounds to chelate transition metal ions such as copper and iron. By sequestering these metals, chelators inhibit their participation in reactions like the Fenton and Haber–Weiss reactions, thereby preventing the generation of highly reactive hydroxyl radicals (49). Antioxidants that are effective in chelating ferrous ions contribute to reducing oxidative stress by forming stable, water-soluble complexes with iron. These complexes facilitate the mobilization and elimination of excess iron from tissues, allowing it to be excreted safely through urine and faeces (43). The IC₅₀ value for metal chelating activity for bael gum was found to be 765.7 ± 1.5 mg/g. Comparatively, previous research has reported varying antioxidant potentials across different parts of the bael plant. For instance, a study by Wali et al. (50) evaluated methanolic extracts of bael leaf, bark, and fruit. The leaf extract exhibited the highest DPPH radical scavenging activity with an IC₅₀ value of 249.3 ± 9.4 μg/mL, while the fruit extract showed the least activity with an IC₅₀ of 1032.2 ± 7.03 μg/mL. In terms of metal chelating activity, the leaf extract again outperformed the others, with an IC₅₀ of 165.7 ± 2.3 μg/mL, compared to 977 ± 5.7 μg/mL for the fruit extract.

Phenolic compounds are well-recognized for their antioxidant properties, contribution to human health (51). In light of this, the total phenolic content of bael gum was evaluated and found to be 90.3 ± 1 μg/g, indicating a notable presence of antioxidant constituents. Similarly, Yadav et al. (17) reported that Chironji gum exhibited substantial antioxidant activity, including significant DPPH and ABTS radical scavenging capacities, as well as metal chelating ability, further supporting the relevance of gum-derived phenolics in antioxidant defense.

### 3.5 Antidiabetic assay

The digestive enzymes *α*-amylase and *α*-glucosidase play essential roles in the hydrolysis of complex carbohydrates into glucose, enabling its absorption into the bloodstream. Inhibiting these enzymes can effectively slow carbohydrate digestion and reduce postprandial hyperglycemia, offering a therapeutic strategy for managing type 2 diabetes (52). Several studies have reported that phytochemicals, particularly phenolic compounds, have inhibitory effects on *α*-amylase and α-glucosidase, and may be useful in treatment of diabetes (53, 54). In addition, plant-derived antioxidants can shield *β*-cells from. Reactive Oxygen Species (ROS), which may help prevent diabetes brought on by ROS (55). Consequently, it is essential to explore natural sources that could be used to lower blood glucose levels. In this study, the *in vitro* antidiabetic potential of bael gum was evaluated by assessing its inhibitory activity against *α*-amylase and α-glucosidase, expressed as IC₅₀ values. The IC₅₀ values for bael gum extract were found to be 1,126 ± 18 μg/mL for α-amylase and 853 ± 15 μg/mL for α-glucosidase (Table 3), indicating a stronger inhibitory effect on α-glucosidase. These results suggest that bael gum possesses moderate antidiabetic potential and may contribute to the regulation of postprandial blood glucose levels. However, previous studies have reported significantly lower IC₅₀ values for α-amylase and α-glucosidase inhibition in the leaf extracts of *Aegle marmelos* (56, 57), indicating that bael leaves exhibit superior antidiabetic activity compared to bael gum.

### 3.6 UHPLC-QTOF-MS analysis of bael gum

**Figure 3:**
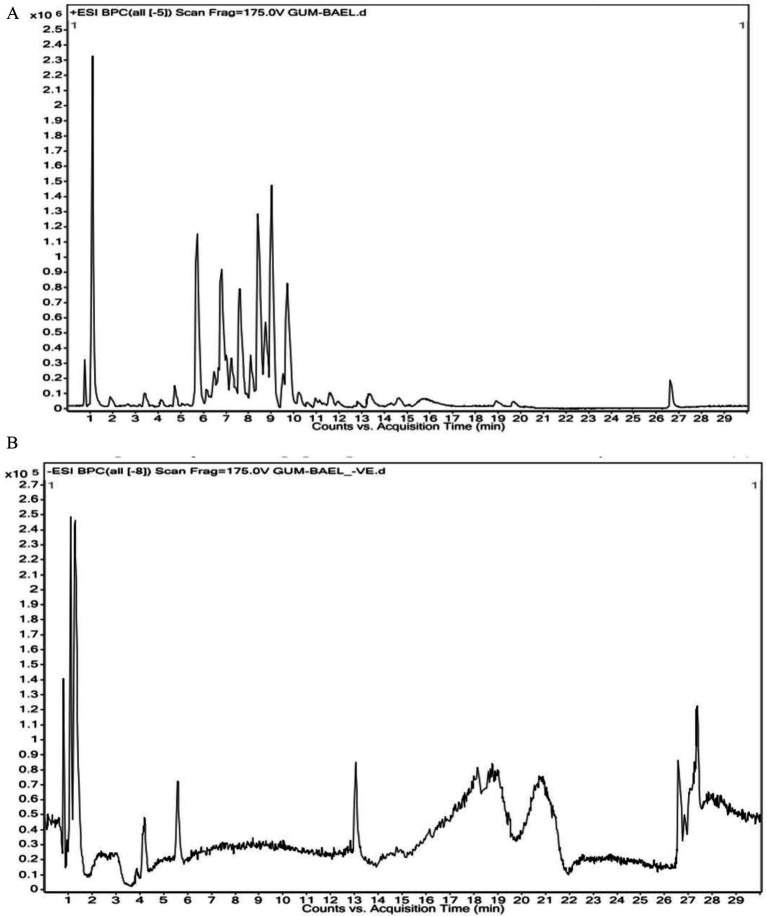
**(A)** HR-LCMS positive chromatogram of bael gum extract showing prominent compound corresponding to retention time. **(B)** HR-LCMS negative chromatogram of bael gum extract showing prominent compound corresponding to the retention time.

**Figure 4:**
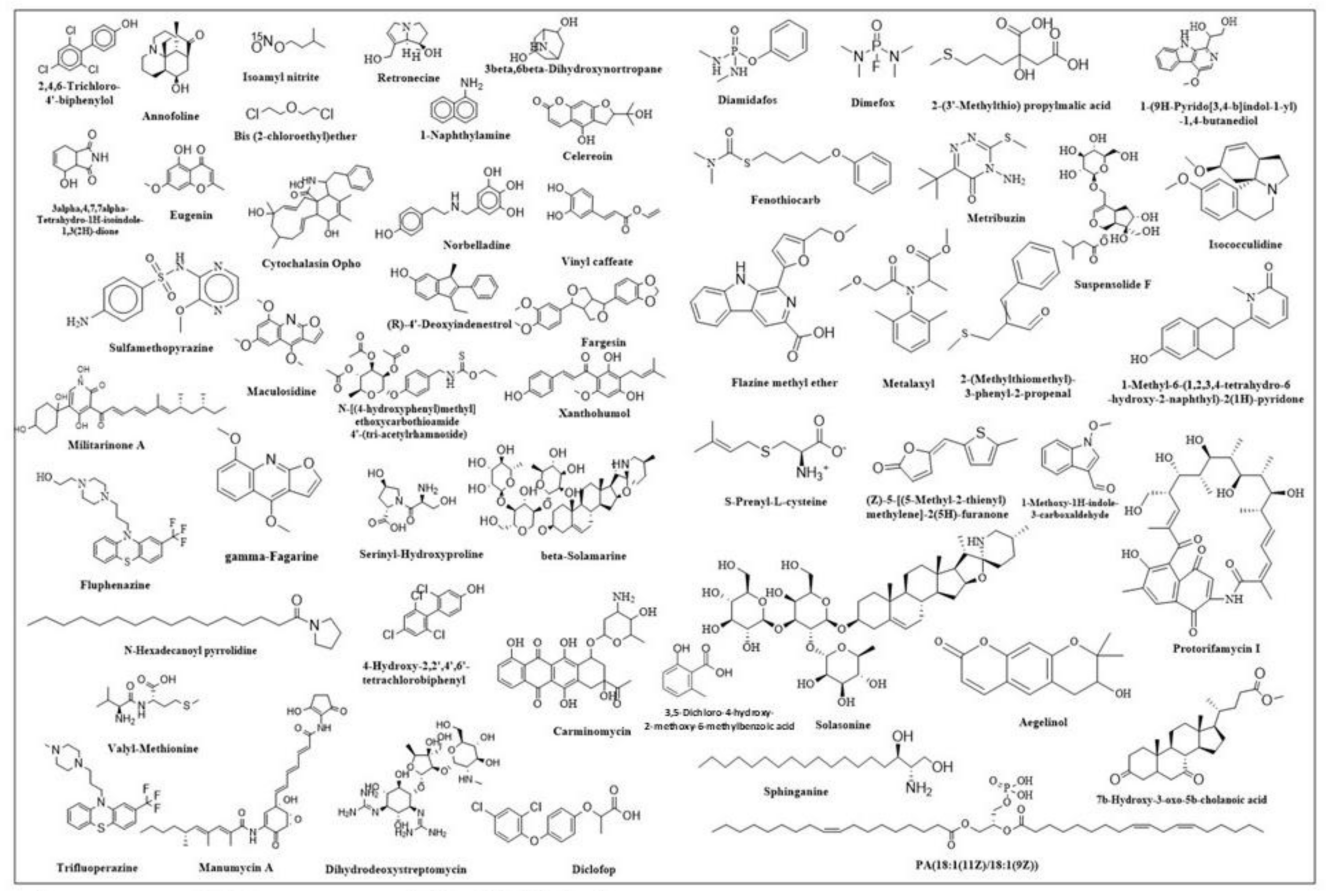
Structure of different compounds identified in bael gum.

The findings of the analysis using liquid chromatography-time of flight mass spectrometry (LC–MS/MS) are shown in Table 4. A total of 71 phytochemicals were identified in the bael gum aqueous extract, comprising 67 in positive mode and 4 in negative mode. The LC–MS/MS spectra are illustrated in Figures 3A,B, while the structures of the identified compounds are depicted in Figure 4. The functions of 17 of the 67 compounds found by positive mode analysis were unknown, whereas the 50 bioactive compounds were divided into various classes such as alkaloids, alkyl group, amine, amyl group, aromatic ether, benzene sulfonamides, carboxylic acids, chalcones, chromones, cinnamaldehydes, coumaric acids, cysteine, dipeptides, dipeptides, furanoquinolines, glycoalkaloid, glycoside, hydroxybiphenyls, indoles, *lignans,* monohydroxy bile acids, n-acylpyrrolidine, o-methoxybenzoic acids, organic compound, organic phosphoramides, phenols, phenothiazine, Polyketides*, proline*, psoralens, *steroidal saponins*, terpene glycosides, thiophenes, and triazines. Table 5 outlines the functions of the found molecule, showing that bael gum has a significant bioactive compound with a variety of applications, including antifungal (*β*-Solamarine, 4-Hydroxy-2,2′,4′,6′-tetrachlorobiphenyl metalaxyl), anti-cancerous (3α, 4,7,7α-Tetrahydro-1H-isoindole-1,3(2H)-dione, Carminomycin, Solasonine, β-Solamarine, Aegelinol, Fluphenazine, Xanthohumol, Serinyl-Hydroxyproline, Manumycin A), antibacterial (Retronecine, Celereoin, Aegelinol, Carminomycin, Dihydrodeoxystreptomycin, Serinyl-Hydroxyproline, Manumycin-A), antimicrobial (Sulfamethopyrazine, Celereoin, N-Hexadecanoyl pyrrolidine, Manumycin-A,), antiviral (Eugenin), antimalarial (Maculosidine, Benzenesulfonamides), acaricide (Dimefox, Fenothiocarb), antioxidant (Retronecine, 1-Naphthylamine, Norbelladine, Celereoin, 1-Methoxy-1H-indole-3-carboxaldehyde, Vinyl caffeate, Aegelinol, Serinyl-Hydroxyproline, 7b-Hydroxy-3-oxo-5b-cholanoic acid), antidiabetic (3*β*,6β-Dihydroxynortropane), anti-atherosclerotic (Manumycin A), antiparasitic (Benzenesulfonamides), anti-inflammatory (β-Solamarine, Fargesin, Norbelladine), antineoplastic (β-Solamarine), and antiprotozoal (β-Solamarine, *γ*-Fagarine) compounds.

**Table 4:** Compounds identified in bael gum by positive and negative mode of analysis.

S. N.	Name	Formula	RT	m/z	Mass	Score	Diff (DB, ppm)	Height
Compound identified through the positive mode of analysis								
1	2,4,6-Trichloro-4′-biphenylol	C_12_H_7_Cl_3_O	0.80	272.96	271.95	60.23	6.68	104084.00
2	Bis (2-chloroethyl) ether	C_4_H_8_Cl_2_O	0.84	143.00	142.00	57.35	−3.86	84331.00
3	Isoamyl nitrite	C_5_H_11_NO_2_	1.05	118.09	117.08	79.56	−9.98	42891.00
4	Retronecine	C_8_H_13_NO_2_	1.20	156.10	155.10	80.57	−11.83	83145.00
5	3β,6β-Dihydroxynortropane	C_7_H_13_NO_2_	1.50	144.10	143.10	88.09	−10.44	48367.00
6	3α,4,7,7α-Tetrahydro-1H-isoindole-1,3(2H)-dione	C_8_H_9_NO_2_	2.67	152.07	151.06	77.78	−8.74	41678.00
7	1-Naphthylamine	C_10_H_9_N	3.43	144.08	143.08	75.11	−10.91	61237.00
8	Cytochalasin Opho	C_28_H_37_NO_4_	4.16	474.26	451.27	93.06	−1.75	45308.00
9	Norbelladine	C_15_H_17_NO_3_	4.77	260.13	259.12	83.88	−8.29	103632.00
10	(R)-4’-Deoxyindenestrol	C_18_H_18_O	4.89	273.13	250.14	91.96	−3.95	61464.00
11	Annofoline	C_16_H_25_NO_2_	5.04	264.20	263.19	80.81	−8.43	43096.00
12	Militarinone A	C_26_H_37_NO_6_	5.19	460.28	459.27	44.99	−18.94	55852.00
13	Dimefox	C_4_H_12_FN_2_OP	5.22	177.06	154.07	79.64	−4.57	41706.00
14	Sulfamethopyrazine	C_11_H_12_N_4_O_3_S	5.59	303.05	280.06	93.42	−0.56	55657.00
15	2-(3′-Methylthio) propylmalic acid	C_8_H_14_O_5_S	5.84	223.06	222.06	84.35	4.28	55440.00
16	Diamidafos	C_8_H_13_N_2_O_2_P	6.20	223.06	200.07	87.84	−8.16	110653.00
17	Celereoin	C_14_H_14_O_5_	6.40	263.09	262.09	74.78	−9.28	42747.00
18	Fenothiocarb	C_13_H_19_NO_2_S	6.52	254.12	253.11	84.36	3.68	64954.00
19	1-(9H-Pyrido[3,4-b]indol-1-yl)-1,4-butanediol	C_15_H_16_N_2_O_2_	6.73	257.13	256.12	78.06	−5.82	44563.00
20	PA(18:1(11Z)/18:1(9Z))	C_39_H_73_O_8_P	6.73	701.50	700.49	52.40	20.03	69478.00
21	Eugenin	C_11_H_10_O_4_	6.79	207.07	206.06	81.29	−10.72	422275.00
22	Solasonine	C_45_H_73_NO_16_	6.90	884.51	883.50	80.58	−5.42	100871.00
23	1-Methoxy-1H-indole-3-carboxaldehyde	C_10_H_9_NO_2_	7.02	176.07	175.07	85.07	−10.39	154370.00
24	β-Solamarine	C_45_H_73_NO_15_	7.06	868.51	867.50	79.80	−5.82	93984.00
25	Vinyl caffeate	C_11_H_10_ O_4_	7.12	207.07	206.06	82.40	−10.26	161800.00
26	Fargesin	C_21_H_22_O_6_	7.24	371.15	370.14	98.72	−0.75	49436.00
27	Aegelinol	C_14_H_14_O_4_	7.24	247.10	246.09	81.34	−9.54	274701.00
28	N-[(4-hydroxyphenyl)methyl] ethoxycarbothioamide 4′-(tri-acetylrhamnoside)	C_22_H_29_NO_9_S	7.49	484.16	483.16	92.36	−0.07	135959.00
29	Metribuzin	C_8_H_14_N_4_OS	7.61	237.08	214.09	92.28	−2.20	436471.00
30	Flazine methyl ether	C_18_H_14_N_2_O_4_	7.85	323.11	322.10	75.77	−8.84	100645.00
31	Isococculidine	C_18_H_23_NO_2_	7.97	308.17	285.18	59.37	−16.64	81253.00
32	Carminomycin	C_26_H_27_NO_10_	8.08	514.17	513.17	85.98	−5.45	82585.00
33	S-Prenyl-L-cysteine	C_8_ H_15_NO_2_S	8.12	190.09	189.08	86.11	6.24	180993.00
34	Trifluoperazine	C_21_H_24_F_3_N_3_S	8.36	430.15	407.16	88.66	1.04	50539.00
35	Fluphenazine	C_22_H_26_F_3_N_3_OS	8.66	460.16	437.17	90.62	1.24	42002.00
36	Maculosidine	C_14_H_13_NO_4_	8.71	260.09	259.09	82.81	−8.86	258869.00
37	Dihydrodeoxystreptomycin	C_21_H_41_N_7_O_11_	9.02	568.30	567.29	62.87	−11.06	312153.00
38	Manumycin A	C_31_H_38_N_2_O_7_	9.04	573.25	550.27	81.26	4.17	51935.00
39	γ-Fagarine	C_13_H_11_NO_3_	9.12	230.08	229.08	85.13	−8.57	83610.00
40	Xanthohumol	C_21_H_22_O_5_	9.17	355.15	354.15	99.04	0.59	78867.00
41	Protorifamycin I	C_35_H_45_NO_10_	9.54	640.32	639.31	42.70	−14.50	47891.00
42	Metalaxyl	C_15_H_21_NO_4_	9.54	280.16	279.15	83.89	−8.10	124351.00
43	Valyl-Methionine	C_10_H_20_N_2_O_3_S	9.81	249.13	248.12	88.61	1.99	77143.00
44	1-Methyl-6-(1,2,3,4-tetrahydro-6-hydroxy-2-naphthyl)-2(1H)-pyridone	C_16_H_17_NO_2_	10.06	256.14	255.13	80.65	−9.48	99135.00
45	Sphinganine	C_18_H_39_NO_2_	11.01	302.31	301.30	84.09	−7.53	73350.00
46	Serinyl-Hydroxyproline	C_8_ H_14_N_2_O_5_	11.14	219.10	218.09	83.95	2.88	60428.00
47	Diclofop	C_15_H_12_C_l2_O_4_	11.33	327.01	326.00	27.74	25.54	52716.00
48	Suspensolide F	C_21_H_34_ O_12_	12.88	501.19	478.21	95.92	0.16	45719.00
49	N-Hexadecanoylpyrrolidine	C_20_H_39_NO	18.94	310.31	309.31	73.46	−8.43	58346.00
50	7b-Hydroxy-3-oxo-5b-cholanoic acid	C_24_H_38_O_4_	19.68	391.29	390.28	71.09	−8.27	57734.00
Compound identified through the negative mode of analysis								
51	4-Hydroxy-2,2′,4′,6′-tetrachlorobiphenyl	C_12_H_6_C_l4_O	0.91	304.91	305.92	62.27	−5.34	151062.00
52	2-(Methylthiomethyl)-3-phenyl-2-propenal	C_11_H_12_OS	1.13	191.05	192.06	91.12	−0.16	129387.00
53	(Z)-5-[(5-Methyl-2-thienyl) methylene]-2(5H)-furanone	C_10_H_8_O_2_S	1.30	191.02	192.02	82.60	−2.15	149961.00
54	3,5-Dichloro-4-hydroxy-2-methoxy-6-methylbenzoic acid	C_9_ H_8_Cl_2_O_4_	20.35	248.97	249.98	52.05	5.52	108168.00

**Table 5:** Function of important compounds identified in bael gum.

Sr. No.	Name	Formula	Class	Functions	Reference
1	Isoamyl nitrite	C_5_ H_11_ N O_2_	Amyl group	Antihypertensive agent	https://pubchem.ncbi.nlm.nih.gov/compound/Amyl-Nitrite
2	3β,6β-Dihydroxynortropane	C_7_ H_13_ N O_2_	Tropane alkaloids	Antidiabetic property	(78)
3	Retronecine	C_8_ H_13_ N O_2_	Pyrrolizidine alkaloid	Antibacterial and antioxidant	(79)
4	3α,4,7,7α-Tetrahydro-1H-isoindole-1,3(2H)-dione	C_8_ H_9_ N O_2_	Isoindolones	Anticancer	(58)
5	1-Naphthylamine	C_10_ H_9_ N	Amine	Antioxidant	https://www.knowde.com
6	Norbelladine	C_15_ H_17_ N O_3_	Amaryllidaceae alkaloids	Anti-inflammatory, antioxidant	(80)
7	Annofoline	C_16_ H_25_ N O_2_	Alkaloids	Antioxidant	(81)
8	Sulfamethopyrazine	C_11_ H_12_ N_4_ O_3_ S	Benzenesulfonamides	Antimicrobial, Antimalarial, antiparasitic	www.ebi.ac.uk
9	Celereoin	C_14_ H_14_ O_5_	Psoralens	Antimicrobial, Antioxidant	(82)
10	Eugenin	C_11_ H_10_ O_4_	Chromones	Antiviral agent (against the main protease of SARS-cov-2)	(66)
11	Solasonine	C_45_ H_73_ N O_16_	Glycoalkaloid	Anti-inflammatory, Anticancerous	(83)
12	1-Methoxy-1H-indole-3-carboxaldehyde	C_10_ H_9_ N O_2_	Indoles	Antioxidants	(84)
13	β-Solamarine	C_45_ H_73_ N O_15_	*Steroidal saponins*	Antineoplastic, antiinflammatory, anticarcinogenic, antiprotozoal (leishmania), antifungal, hepatoprotective	(85)
14	Vinyl caffeate	C_11_ H_10_ O_4_	Coumaric acids	Antioxidants	(86)
15	Fargesin	C_21_ H_22_ O_6_	*Lignans*	Anti-inflammation	(87)
16	Aegelinol	C_14_ H_14_ O_4_	Linear pyranocoumarins	Antibacterial, antioxidant activities and anticancerous	(88)
17	N-[(4-hydroxyphenyl) methyl] ethoxycarbothioamide 4′-(tri-acetylrhamnoside)	C_22_ H_29_ N O_9_ S	Glycoside	Antifungal (Anti oyomycotic)	(89)
18	Carminomycin	C_26_ H_27_ N O_10_	Anthracenecarboxylic acids	Anitcanerous (Antitumor), antibiotic	(90)
19	Trifluoperazine	C_21_ H_24_ F_3_ N_3_ S	Antipsychotics	Antimetastasis agent	(91)
20	Maculosidine	C_14_ H_13_ N O_4_	Organic heterotricyclic	Anti-malarial activity	(92)
21	Fluphenazine	C_22_ H_26_ F_3_ N_3_ O S	Phenothiazine	Anticancer activity	(59)
22	γ-Fagarine	C_13_ H_11_ N O_3_	Furanoquinolines	Antitrichomonal activities	(93)
23	Dihydrodeoxystreptomycin	C_21_ H_41_ N_7_ O_11_	Amino cyclitol & glycoside.	Antibacterial activity	(94)
24	Manumycin A	C_31_ H_38_ N_2_ O_7_	Polyketides	Anticancer, antibiotic, antimicrobial, antiatherosclerotic	www.ebi.ac.uk, pubchem.ncbi.nlm. Nih. gov and medchemexpress.com
25	Xanthohumol	C_21_ H_22_ O_5_	Chalcones	Anticancer	(95)
26	Serinyl-Hydroxyproline	C_8_ H_14_ N_2_ O_5_	*Proline*	Antioxidant, Antibacterial and Anticancer	(84)
27	N-Hexadecanoyl pyrrolidine	C_20_ H_39_ N O	N-acylpyrrolidine	Antimicrobial	(96)
28	7b-Hydroxy-3-oxo-5b-cholanoic acid	C_24_ H_38_ O_4_	Monohydroxy bile acids	Antioxidants	(97)
29	4-Hydroxy-2,2′,4′,6′-tetrachlorobiphenyl	C_12_ H_6_ C_l4_ O	Hydroxybiphenyls	Antifungal	(98)

Globally, cancer is predicted to claim the lives of 10.0 million people, and 19.3 million more cases are expected in 2020. Globally, 18,094,716 instances of cancer were reported in 2020. The rate was higher for men (206.9 per 100,000) than for women (178.1 per 100,000). Preventing cancer is therefore a major public health concern because it is getting increasingly prevalent in practically every nation. By addressing risk factors related to nutrition, exercise, and food, almost 40% of cancer cases could be prevented. Nine phytochemicals with anti-cancer properties were found in bael gum, including 3α,4,7,7α-Tetrahydro-1H-isoindole-1,3(2H)-dione, Carminomycin, Solasonine, β-Solamarine, Aegelinol, Fluphenazine, Xanthohumol, Serinyl-Hydroxyproline, and Manumycin A (58, 59). Therefore, bael gum might be a crucial source for the extraction of anti-cancer compounds. According to reports, the Arabic gum of *Acacia Senegal* (60), guggulipid of *Commiphora mukul* (61), *Boswellia sacra* gum samroresins (62), asafoetida gum (63), *Azadirachta indica* gum (64) and Chironji gum (17) also contains anti-cancerous phyto-chemicals.

The human population is currently infected with 219 different types of viruses, and the world has experienced multiple viral disease pandemics in the past that have not only negatively impacted human life (65) but also hampered economic growth and development, as well as other industries like agriculture, animals, and poultry. The documented viral pandemics include SARS-CoV-1 (Severe Acute Respiratory Syndrome) from 2002 to 2004, Influenza A H1N1 (Swine Flu) in 2009, Middle East Respiratory Syndrome (MERS) CoV Infection in 2012, the Ebola Virus Pandemic from 2013 to 2016, the Zika and SARS-CoV-2 epidemics in 2015, and the SARS-CoV-2 Pandemic from 2019 to 2022. In order to treat viral infections, new bioactive chemical sources must be investigated due to the global population pressure. In this context, we have also discovered Eugenin, an antiviral drug that works against SARS-CoV-2’s primary protease in the bael gum (66). Therefore, the bael gum may be essential for controlling SARS-CoV-2.

Fungal diseases, which are already a major global concern, are becoming worse for both humans and plants and are responsible for about 1.7 million fatalities each year. Only a few hundred of the 1.5 to 5.0 million fungal species (67), can infect healthy people and cause illness in humans (68, 69). Three antifungal chemicals, *β*-solanine, 4-Hydroxy-2,2′,4′,6′-tetrachlorobiphenyl, and Metalaxyl, have been found in bael gum. The Metalaxyl present in bael gum may have been transferred from the soil as a result of an earlier application made during the planting stage to manage the fungal disease in bael. Similarly, antifungal compounds have also been reported in different plants of gum, like mastic tree gum, Chironji gum, and asafoetida gum (17, 63).

Antioxidants are extensively used in cosmetics, medicines, and food processing industries. Because of their anti-aging properties and capacity to manage neurological diseases, diabetes mellitus, cancer, and anti-inflammatory diseases (70). People nowadays are more curious about having a diet rich in antioxidants. Besides this, they are also used in encapsulation to prevent spoilage and stabilize foods. In the present study, we have found nine antioxidant compounds in bael gum (Retronecine, 1-Naphthylamine, Norbelladine, Celereoin, 1-Methoxy-1H-indole-3-carboxaldehyde, Vinyl caffeate, Aegelinol, Serinyl-Hydroxyproline, Annofoline, and 7b-Hydroxy-3-oxo-5b-cholanoic acid), which can be used for different purposes such as food processing, cosmetics, pharmaceuticals, and other uses. Similarly, antioxidants have also been reported in different plant gums, such as neem (64), *Cordia myxa* (71), *A. Senegal* (72), guggul (73) and Chironji (17). The presence of an antidiabetic compound, i.e., 3*β*, 6β-Dihydroxynortropane, identified through phytochemical screening, supports the results of our anti-diabetic assay, indicating that bael gum can play a crucial role in preventing diabetes. The analysis of bael gum revealed the presence of several other bioactive compounds exhibiting diverse therapeutic potentials. Notably, it contains Manumycin A, known for its anti-atherosclerotic activity, which helps in preventing the formation of arterial plaques (74). The presence of Benzenesulfonamides indicates antiparasitic properties, suggesting potential use against parasitic infections (75). Compounds such as β-Solamarine, Fargesin, and Norbelladine contribute to the anti-inflammatory activity (76), while β-Solamarine also demonstrates antineoplastic effects, highlighting its possible role in cancer prevention or treatment. Furthermore, both β-Solamarine and *γ*-Fagarine exhibit antiprotozoal activity (77), supporting the traditional medicinal use of bael in managing protozoal infections. Overall, these findings underscore bael gum’s multifaceted pharmacological potential and its relevance in developing natural therapeutic agents.

The present study profiled bael gum using samples collected through random sampling, which provided an overall understanding of its biochemical and nutraceutical attributes. However, this approach does not account for the substantial variability that may occur among individual plants, environmental conditions, plant age, management practices, or seasonal influences. For future investigations, quantifying gum yield per plant, evaluating nutraceutical and bioactive compound variability across different genotypes and environments, and developing standardized protocols for gum extraction and characterization will be essential. Incorporating these aspects will enhance the robustness, reproducibility, and applicability of the findings, thereby strengthening the scientific value and practical relevance of bael gum research.

## 4 Conclusion

The findings of this study demonstrate that bael gum possesses nutraceutical and therapeutic potential, primarily due to its rich composition of antioxidants, antioxidant enzymes, and diverse bioactive compounds. These attributes make it a promising candidate for use as a natural food additive and functional ingredient. The presence of bioactive chemicals beneficial to both plant and human health suggests broad applicability in industries such as food, dairy, beverage, cosmetic, plant protection, and pharmaceutical. This study offers a novel and comprehensive insight into the phytochemical screening of bael gum and establishes it as an imperative source of compounds with the potential to cure a variety of human ailments. Given these promising results, future research should aim to conduct *in vivo* studies and clinical trials to validate the health benefits observed *in vitro*. Additionally, exploring the mechanisms of action of specific bioactive components and assessing their efficacy in functional food and therapeutic formulations will be crucial for advancing the practical applications of bael gum.

## Data Availability

The data presented in the study are deposited in the Figshare database and is available on following link: https://doi.org/10.6084/m9.figshare.30656966.

## Footnotes

1
https://www.flowersofindia.net/catalog/slides/Bel.html
